# Exploring Barriers and Enablers for the Intention to Use Assistive Robotics Among People With Spinal Cord Injury and Those Involved in Their Care: Qualitative Study

**DOI:** 10.2196/72080

**Published:** 2026-02-17

**Authors:** Susanne Frennert, Johanna Persson, Eva Díez-Rodríguez, Monica Alcobendas-Maestro, Fátima Villamayor Vega, Antonio Oliviero

**Affiliations:** 1 Department of Design Sciences, Division of Ergonomics and Aerosol Technology, Ingvar Kamprad Designcentrum (IKDC), Lund University, FormStråket 12, Lund, 223 62, Sweden, 46 703876838; 2 FENNSI Group, Hospital Nacional de Parapléjicos and “Instituto de Investigación Sanitaria de Castilla-La Mancha” (IDISCAM), SESCAM, Toledo, Spain

**Keywords:** barriers, enabler, assistive robotics, spinal cord injury, spinal cord injury care

## Abstract

**Background:**

Spinal cord injury (SCI) may, and often does, profoundly reshape daily life, altering physical abilities, social roles, and personal identities. While assistive technologies, including assistive robotics, are often framed as solutions to re-establish independence, their adoption is shaped by practical, emotional, and social considerations as well as functional qualities. Individuals with SCI, their relatives, and health care professionals need to navigate complex dynamics when encountering assistive robotics. Understanding how assistive technologies are perceived and positioned in everyday life may help developers and designers create assistive robotics that are meaningful and useful for intended users.

**Objective:**

The aim of this qualitative study was to explore how individuals with SCI, relatives, and

health care professionals working with patients with SCI perceive and describe the possibilities and limitations of assistive robotics. The study sought to understand the factors that influence the intention to use assistive robotics among individuals with SCIs.

**Methods:**

We used a qualitative approach, conducting semi-structured interviews and participatory workshops in Sweden and Spain. In total, the study involved 18 interview participants with SCI, 21 workshop participants with SCI, 12 relatives, and 26 health care professionals. The interviews and workshops elicited reflections on participants’ experiences, expectations, and concerns regarding assistive robotics in general and supernumerary robotic limbs in particular. Data were analyzed using reflexive thematic analysis, with a focus on interpreting the meanings embedded in participants’ narratives.

**Results:**

The analysis showed that participants’ engagement with assistive robotics was influenced by expectations of technological benefits and by practical constraints in everyday life. The main barriers identified were practical constraints, including the subthemes “navigating a changing reality,” “difficulties with awareness and access” and “concerns about costs”; and interaction with robots, including “doubts about meaningfulness,” “uncertainty regarding reliability and safety,” “uneasiness about competence” and “apprehension of social norms.” Participants’ visions of enhanced self-efficacy through assistive robotics were described as important enablers of the intention to use and motivation to try assistive robotics. Shared expectations and concerns about future technologies (technological imaginaries) also influenced how participants talked about assistive robotics.

**Conclusions:**

Rather than presenting assistive robotics as an inevitable progression toward greater autonomy, this study highlights the complexities and contingencies that shape how individuals relate to assistive robotics in general and supernumerary robotic limbs in particular. Participants’ responses illustrate that robotic assistance is not merely a question of technological feasibility but is deeply entangled with embodied experiences, shifting identities, and evolving social relations. While visions of independence through assistive robotics remain compelling among participants, sociotechnical imaginaries coexist with concerns about meaningful engagement, reliability, safety, competence, and social norms, as well as challenges related to transition periods, costs, and limited awareness and access to assistive robotics.

## Introduction

Spinal cord injury (SCI) has a substantial impact on quality of life, affecting physical, psychological, social, and economic aspects of an individual’s life. The severity and level of the injury influence the extent of this impact, often resulting in paralysis or loss of sensation and a need for extensive assistance in everyday life [1]. This, in turn, negatively affects the ability of individuals with SCI to independently perform everyday tasks, such as hygiene, eating, and dressing. Frequently, they depend on assistance from caregivers, relatives, and various assistive aids [2].

In robotics labs around the world, several projects are being conducted to develop valuable robotic assistance for people with SCI, such as mobile manipulators [3] and robot-assisted feeding [4]. In this paper, we use the term “assistive robotics” to refer to robotic devices that provide physical assistance for everyday activities to people with upper body-limb disabilities (eg, robotic arms, powered gloves, and exoskeletons that support feeding, reaching, dressing, or grooming).

However, despite the potential benefits of assistive technologies, abandonment rates remain high [5], often due to technology-driven design that overlooks the social and cultural factors affecting user acceptance [6]. A key factor influencing user abandonment is the intended users’ perception of the technology in question [7-9].

### Conceptual Framework and Research Gap

The perception of technology and its relation to the intention to use are explained by the Technology Acceptance Model (TAM) [10] and the Unified Theory of Acceptance and Use of Technology (UTAUT) [11]. TAM focuses on personal beliefs about usefulness and ease of use [10], while UTAUT adds social influence and the broader conditions surrounding the use of technology [11]. In the context of individuals with SCI, the models suggest that the intentions to use assistive technologies are influenced not by personal beliefs but also by the views and practices of caregivers, relatives, and health care professionals (HCPs) who organize care and everyday use [12].

At the same time, TAM and UTAUT were developed for information systems and “able-bodied individuals” [10,11], and may therefore not fully capture the situated, embodied, and relational aspects of technology adoption in a disability context. Building on these models, work in assistive technologies has emphasized the importance of perceived meaningfulness, identity, and the organization of support around assistive technologies [7,9,13,14]. Concepts from Science and Technology Studies such as sociotechnical imaginaries draw attention to shared visions and narratives about how emerging technologies might reshape care and independence [15-19]. Sociotechnical imaginaries highlight how hopes, fears, and expectations about technologies are collectively produced.

Existing research on assistive robotics and people with SCI has primarily addressed rehabilitation and technical feasibility. Studies have investigated robots for rehabilitation [20-22], work participation [23,24], technology use and priorities [25,26], outcome measures [27] and user-centered design guidelines [28]. Several recent systematic reviews and state-of-the-art papers synthesize the development of upper-limb assistive and rehabilitation robots, including exoskeletons, end-effector devices, soft exosuits for people with cervical SCI and other neuromuscular conditions [29-33]. The reviews describe control strategies, clinical indications, motor outcomes, and identify challenges, such as comfort, wearability, and robustness. Furthermore, a systematic review has examined the barriers and facilitators to exoskeleton use [34] and broader acceptance of assistive technologies among people with motor disabilities [9].

Across the existing literature, user perspectives are most often operationalized in terms of usability, comfort, and short-term feasibility, focusing on the person undergoing rehabilitation [9,29,32]. While some studies included HCPs and, more rarely, relatives as respondents, they are not systematically treated as stakeholders, even though they are often responsible for procuring assistive technologies, providing training, and embedding them in care routines [34]. There are also very few empirical studies on how emerging technologies such as supernumerary robotic limbs (SRLs) are imagined before they reach the market [30,33], which leaves a gap in understanding how assistive robotics are positioned within the everyday lives of people with SCI and those involved in their care.

To address the identified gap, this study is conducted within the HARIA project, which develops SRLs to augment upper-limb function in people with substantial arm and hand impairments [35]. SRLs are wearable robotic arms and fingers, designed to enhance human sensorimotor abilities, particularly in tasks involving movement and coordination [36]. For individuals with SCI, SRLs are proposed as aids to compensate for lost functions in impaired limbs, provide necessary assistance, and substitute for impaired limbs in terms of functionality [37].

### Aim and Research Question

The objective of this qualitative study was to explore how individuals with SCI, their relatives, and HCPs conceptualize, engage with, and negotiate the possibilities and limitations of assistive robotics in general, with a particular focus on SRLs. More specifically, we examined (1) intentions to use emerging assistive robotics that are not yet widely available, (2) how these intentions are shaped by experiences of both used and abandoned assistive technologies, and (3) how perspectives differ or converge across stakeholders, including individuals with SCI, relatives, and HCPs in Sweden and Spain.

We asked: What barriers and enablers shape the intention to use assistive robotics among people with SCI and those involved in their everyday care in Sweden and Spain? By exploring this question, we aimed to clarify how assistive robotics are positioned within the everyday realities of potential users and their caregivers and to inform the design and implementation of emerging assistive robotics.

## Methods

### Study Design

**Figure 1.**
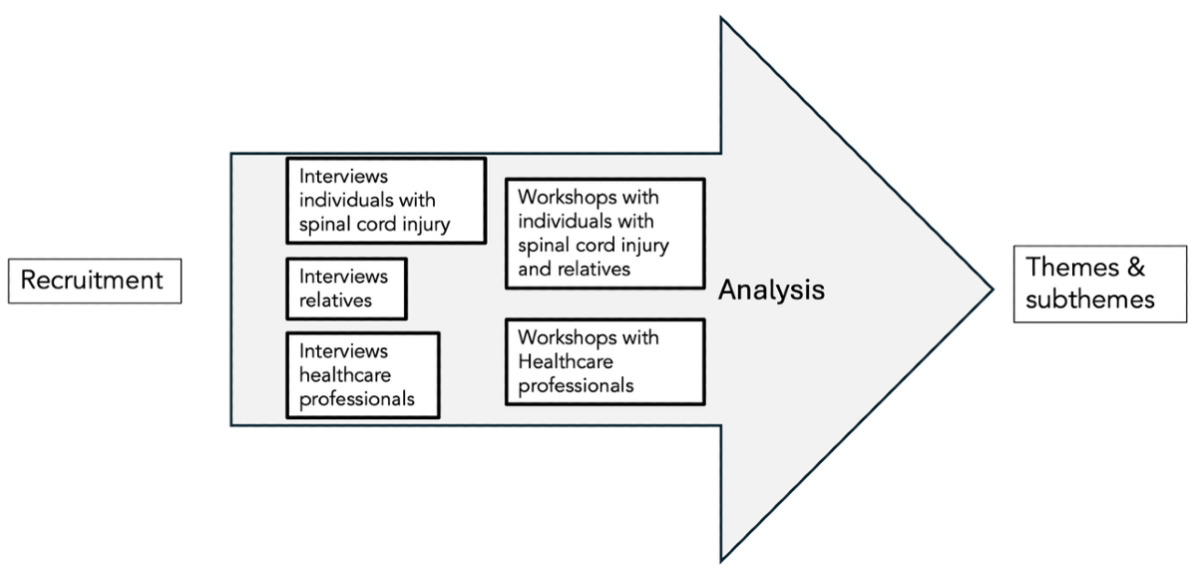
Study design.

This qualitative exploratory study is part of a broader research project centered around SRLs [35,37-39]. We adopted an interpretive, constructivist approach [40] and used reflexive thematic analysis [41] to explore how different stakeholders make sense of assistive robotics in the context of SCI. Within this study, our focus is on individuals with SCIs and their intention to use assistive robotics, with a particular emphasis on SRLs. We combined individual semi-structured interviews [42] with participatory workshops [43] to capture both in-depth personal narratives and group-based discussions and co-imaginings of future assistive robotics (Figure 1).

The multidisciplinary research team included researchers in rehabilitation engineering, design science and technology studies, and clinicians, several of whom had prior experience of SCI rehabilitation and assistive technology research. The diversity of the research team informed the development of the interview and workshop guides and the interpretation of the data.

### Setting and Participants

To understand the social dynamics and contextual factors pertinent to assistive robotics, our participant pool included not only individuals with SCI but also HCPs specializing in SCIs, professionals working closely with individuals affected by SCIs, as well as relatives and caregivers. The study was conducted in both Spain and Sweden. In Sweden, participant recruitment involved collaboration with Paraplegic Associations, using their networks to disseminate information about the study to potential participants. Additionally, outreach efforts were extended to various health care facilities specializing in SCIs. In Spain, recruitment targeted HCPs, individuals with SCIs, and relatives through collaboration with Sescam - Gobierno de Castilla-La Mancha, a hospital and rehabilitation facility specializing in SCIs, which facilitated the identification and inclusion of relevant participants.

We focused on adults with cervical SCI because SRLs in the HARIA project aim to augment arm and hand function [35]. We also included relatives and HCPs, based on the assumption that the intention to use assistive robotics is affected by those who support and care for patients with SCI.

### Eligibility and Sample

We contacted potential participants through email and phone. From this pool, we selected a purposive sample by applying specific criteria. To qualify for participation, individuals needed to fulfill the following criteria: (1) be 18 years or older, (2) have acquired a cervical SCI as an adult, (3) live in the community (not in long-term institutional care), and (4) be able to participate in an interview or workshop in Swedish or Spanish. Eligibility extended to those who were either a relative of an individual with a SCI or HCPs working with patients with SCI.

We aimed for heterogeneity to capture a wide range of lived experiences, needs, and expectations. Therefore, we did not set inclusion and exclusion criteria regarding time since injury, lesion level within the cervical region, functional level, or previous experience with assistive technologies or robotics. None of the participants had prior hands-on experience with SRLs, as SRLs were introduced by the researchers during the workshops (Multimedia Appendix 1).

Participants were recruited between January 2023 and September 2023. Recruitment was discontinued when we considered that data saturation was reached, indicating that further interviews produced repetitive information and no new themes or responses emerged [41].

### Data Collection

Data were gathered through audio-recorded qualitative semistructured interviews [42,44] and participatory workshops [43]. The first author formulated the interview and workshop guides (MultimediaAppendices 1^5^), subsequently refining them in collaboration with the rest of the research team [42,45].

The interview questions probed into the experiences of individuals living with SCIs, exploring their current use of assistive technologies and those received but not used (Multimedia Appendix 2). Such questions were included to situate participants’ views on robotics within their broader histories of adopting, adapting to, and sometimes abandoning assistive technologies. Later during the interview, the focus shifted to assistive robotics technology, with questions aimed at understanding the interviewees’ experiences with assistive technologies. During the latter part of the interview, the interviewer provided a brief introduction to SRLs, as the interviewees lacked prior knowledge, and SRLs are not yet commonly available on the market but are currently under development in research laboratories around the world [36,37,46]. Preferences and ideas regarding the use of assistive robotics technology were explored by asking questions about the functions requested, the required design for actual use, and how the interviewees would describe their needs and wishes concerning assistive robotics.

Interview questions and workshop activities for relatives and HCPs followed the same overall structure as for individuals with SCIs but were designed to explore their insights into the needs of the end-users (individuals with SCI) from their relational and professional standpoints (MultimediaAppendices 3^5^). Our questions did not delve into the requirements of relatives and HCPs to acquire, use, or prescribe robotic devices; instead, we focused on their perceptions of the needs of end-users (individuals with SCI). Throughout the sessions, the researchers maintained a field diary. The diary served as a repository for initial analytical reflections, interpretations, and first impressions.

Qualitative semi-structured interviews were conducted either over the phone or digitally by the first [SF] and second [JP] authors in Sweden and by the third [ED], fourth [MA], and fifth [FV] authors in Spain, each lasting approximately 45 to 75 minutes. In total, 18 individuals with SCI, 10 relatives, and 13 HCPs were interviewed.

In the participatory workshops [43], the initial part focused on the lived experiences of individuals with SCIs, identifying both opportunities and challenges (Multimedia Appendix 1). The initial part of the workshop also covered currently used assistive technologies and those left unused. The subsequent part of the workshops centered on SRLs. Here, we showed both illustrations of different scenarios in which SRLs were used and videos of users interacting with SRLs (Multimedia Appendix 1). The same visual material was used across workshops to support comparability between groups of stakeholders and countries. The participants were asked to openly discuss their perceptions of the different scenarios, including perceived barriers and enablers associated with using SRLs. The participatory workshops, with an average duration of 2 hours, included one researcher as the workshop moderator and another responsible for comprehensive notetaking [43]. Upon conclusion, all materials and participant notes were collected.

Two in-person workshops were held in Sweden at Lund University by the first [SF] and second [JP] authors, each lasting around 2 hours. In Spain, 3 in-person workshops were conducted by the third [ED], fourth [MA], and fifth [FV] authors at Sescam - Gobierno de Castilla-La Mancha, a specialized hospital and rehabilitation facility for patients with SCI. Workshops were conducted in small groups and included individuals with SCI and relatives and, in separate workshops, HCPs working in SCI rehabilitation. In total, 21 individuals with SCI, 2 relatives, and 13 HCPs took part in participatory workshops in Sweden and Spain (Table 1).

**Table 1.** Participants in the qualitative semistructured interviews and participatory workshops.

Category	Qualitative semistructured interviews	Male	Female	Age (years), mean	Participatory workshops	Male	Female	Age (years), mean
Participants with spinal cord injury	18	14	4	61	21	16	5	51
Relatives	10	3	7	N/A^a^	2	1	1	N/A
HCPs	13	1	12	N/A	13	2	11	N/A

a Not available.

### Data Analysis

The interviews were transcribed verbatim and subjected to thematic analysis following Clarke and Brown [41]. Initially, the first and the second author individually reviewed the transcripts, immersing themselves in the data through multiple readings. During the initial stage, the researchers systematically assigned codes to expressions and narratives related to barriers and enablers to the intention to use assistive robotics technology. Subsequently, the first and the second author compared codes and resolved any disagreements. The codes were organized into potential key themes and subthemes [41].

Fieldnotes and material from the workshops were analyzed using the same coding framework as the interview transcripts [41]. The shared coding framework allowed us to triangulate across data sources and explore how the same issues were discussed in one-to-one interviews and in group settings (ie, during workshops). Where workshop data introduced new nuances, the coding framework was adjusted and reapplied to interview transcripts.

During the interpretive coding phase, barriers and enabler themes were developed inductively. Initial codes captured a broad range of practical hindrances, concerns, and hopes related to assistive robotics. The codes were gradually organized into categories that reflected patterns across the data (Table 2 presents the final set of themes and subthemes).

For the purpose of verification, workshops dedicated to member checking were organized. Member checking is a qualitative research practice used to ensure data accuracy and credibility by actively involving participants in the data analysis process [47-49]. During the member checking workshops, research findings were presented to individuals with SCIs, their relatives, and HCPs for feedback. The member checking workshops facilitated the refinement of themes to ensure close alignment with participants’ lived experiences of SCI. Quotations are used to illuminate the themes.

**Table 2.** Identified barriers and enablers.

Themes	Subthemes
Barriers	
Practical constraints	Navigating a changing reality Difficulties with awareness and access Concerns about costs
Interactions with assistive robotics	Doubts about meaningfulness Uncertainty regarding reliability and safety Uneasiness about competence Apprehension of social norms
Enablers	
Sociotechnical imaginaries	Imaginings of greater self-efficacy Imaginings of fulfillment of functional requirements

### Ethical Considerations

Ethical approval for this study was obtained from the Swedish Ethical Review Authority (Dnr 2022-03154-01) and Comité Ético CHT Eic-chto-secretario at Toledo Hospital in Spain (CEIC-961). The study was conducted in accordance with the Declaration of Helsinki. All participants provided written informed consent before their participation. Participants were informed in advance that they would not receive any monetary compensation for their time and inconvenience and offered no direct personal benefits. Confidentiality and anonymity of the data provided were maintained throughout the research and publication process.

## Results

### Sample Characteristics

In total, 41 individuals participated in interviews and 36 in participatory workshops (Table 1). Participants were recruited from 2 countries (Sweden and Spain) and 3 stakeholders’ groups (individuals with SCI, relatives, and HCPs). Among participants with SCI, most were men in their fifties and sixties (mean age 61 y in interviews and 51 y in workshops), all with cervical injuries and varying functional levels (ie, most used wheelchairs and required different degrees of assistance with everyday activities such as eating and dressing). Relatives were mainly women, typically partners and close family members. The HCPs were predominantly women and represented a range of professions working with SCI rehabilitation (eg, physiotherapists, rehabilitation physicians, and biomedical engineers).

While the national health care systems differed, for example, in Sweden, participants relied to a greater extent on personal assistants who are paid by the municipalities [50], whereas in Spain, participants with SCIs often depended on their nonpaid relatives [51], similar themes emerged across the 2 country contexts. Such contextual differences, therefore, mainly function as a backdrop for the findings rather than as a primary focus of comparison.

### Overview Themes

In our interpretive reading of the material, the data revealed that participants frequently described barriers impeding their intention to use assistive robotics, both generally and specifically in the context of SRLs. Through reflexive thematic analysis, we organized the data into 2 main barrier themes, “practical constraints” and “interactions with assistive robotics,” and 1 main theme capturing enablers of intention to use “imaginings of self-efficay and functional fulfilment.” Practical constraints include (1) navigating a changing reality, (2) difficulties with awareness and access, and (3) concerns about costs. Interactions with assistive robotics include (1) doubts about meaningfulness, (2) uncertainty regarding reliability and safety, (3) uneasiness about competence, and (4) apprehension of social norms. The main enabler theme encompassed two subthemes: (1) imaginings of greater self-efficacy and (2) imaginings of fulfillment of functional requirements. Key themes and subthemes are represented in Table 2.

### Barrier – Practical Constraints

Overall, 3 subthemes related to barriers associated with practical constraints were identified: “navigating a changing reality,” “difficulties with awareness and access,” and “concerns about costs.” The barrier themes were voiced across all stakeholder groups, although the specific experiences they referred to sometimes differed between individuals with SCI, relatives, and HCPs.

### Navigating a Changing Reality

The subtheme, “navigating a changing reality,*”* captures how participants described ongoing changes over time as a result of the injury, aging, fluctuating symptoms, and evolving technologies as a barrier to engaging with assistive robotics. Most participants, including individuals with SCIs, relatives, and HCPs, described living with a SCI as a constant experience of transition periods. Transitions encompassed both the long-term adjustment to altered functionalities and needs and short-term fluctuations in pain and fatigue. Participants illustrated these experiences in expressions, such as

*It was a brutal impact … from white to black. It shocked me in absolutely everything; it changed my life overnight. Everything has changed*.

[SCI_6]

*As a person with a SCI, you experience neurogenic pains that persist. Some days, it’s really tough.*

[SCI_15]

Those with SCIs also felt that immediately after the accident, they received considerable rehabilitation and assistance at the hospital, but upon discharge, they were largely on their own:

*But now, after being discharged, it’s difficult... no one is keeping track of me...*

[SCI_4]

Such transition periods became a barrier to the intention to use assistive technology as needs, energy levels, and abilities fluctuated throughout the day and changed over time. Consequently, participants were less willing to invest time and energy in assistive technology due to the constant changes, along with fatigue and pain.

Within this subtheme, HCPs frequently cited a barrier to their intention to adopt assistive robotics. They pointed out that rapid technological developments impacted their work, for example, by requiring training in new devices, adapting clinical routines to new technology, and troubleshooting technology during already time-constrained consultations, which they perceived as increasing workload. The continuous changes and advancements placed a strain on them and their engagement with advanced technology. HCPs referred to transition periods in adapting to new technology and the continuous need to learn how to use it. As 1 HCP stated:

*As a disadvantage, it might seem that we would have to learn and train ourselves on these new devices*.

[HCP_18]

Overall, the experience of transitions and constant changes acted as barriers to the intention to use assistive robotics technology.

### Difficulties With Awareness and Access

The subtheme, “difficulties with awareness and access,” illustrates the barriers individuals with SCIs encounter when interacting with HCPs and others, both in understanding their injury’s impact on their lives and in discovering available assistive aids. The subtheme was mainly articulated by individuals with SCI and their relatives, with HCPs offering a partly contrasting perspective on the availability of assistive robotics.

The obstacles outlined by individuals with SCIs included the lack of knowledge among municipality workers, reliance on specialized care professionals, and the challenge of self-identifying their needs:

*Municipal workers lack knowledge about SCIs…we must have availability to direct contact with neurorehabilitation for assistive device support. We as patients typically need to identify our problems and propose potential aids, which may then be either adapted or developed for our individual needs. However, there is no predefined ’catalog’ for selection*.

[SCI_3]

Many participants emphasized the lack of support and assistance regarding assistive aids and knowledgeable personnel. They highlighted this by narrating

*Above all, there is a lack of support on the issue of rehabilitation, there should be more means and more availability, both in terms of aids and personnel*.

[SCI_5]

*…I actually don’t know who I would turn to if I wanted something [aids]*.

[SCI_12]

*I don’t know of any aid. As for support, there isn’t much…*

[SCI_8]

In contrast, HCPs perceived the availability of numerous assistive technologies. However, many also stressed that they did not recommend or prescribe them:

*There are a lot of aids. For example, to eat and drink there are devices that I don’t remember the name of because I have never recommended them*.

[HCP_11]

In general, most participants with SCIs did not rely on advanced assistive technology but on other humans such as relatives and personal assistants. Their dependence on assistive technology mainly centered on wheelchairs and some specialized aids for the bathroom and kitchen. When it came to more advanced assistive technology and robotics, only a limited number of the participants (n=8) had experiences through participating in trials, testing at rehabilitation facilities, or using smart home technology with voice commands to control lighting, TV, and radio.

### Concerns About Costs

Participants with SCI commonly faced financial pressure due to the inability to work or being limited to part-time employment. The subtheme, “concerns about costs,” emphasizes a significant barrier to the intention to use assistive robotics technology tied to financial constraints. Concerns about the affordability of assistive technologies were raised by individuals with SCI, relatives, and HCPs.

In Sweden, known for subsidized health care [50], individuals with SCIs highlighted, like their Spanish counterparts, that only some assistive aids are subsidized [51]. Obtaining advanced assistive technology for free was perceived as challenging and almost impossible.

Some participants were aware of assistive robotics technology through the media but mentioned that trying it out required paying for it themselves. They perceived themselves as a disadvantaged group due to a lack of income and expressed the opinion that having money would make everything easier. For example, 1 participant stated,

*Well, the truth is that I do not have much. Around here, we have a saying that “with money” everything is easier. When you have money, you can afford everything, but if you can’t pay, you will have nothing*.

[SCI_11]

Another participant expressed concerns, stating,

*In terms of support and aids, I think much is missing. Initially, you receive rehabilitation and physiotherapy support, but there comes a time when you either pay out of pocket or receive nothing*.

[SCI_18]

HCPs and relatives also shared concerns about costs, viewing them as a barrier to accessing more advanced assistive technology and human support. When discussing SRLs, participants perceived them as expensive and beyond the scope of ordinary prescribed assistive technologies. As 1 HCP noted,

*The aids must be very affordable so that patients can easily access them*.

[HCP_25]

The cost of assistive technology was consistently perceived as a major barrier affecting the intention to use assistive robotics technology among most of the participants.

### Barrier – Interaction With Assistive Robotics

While practical constraints are linked to tangible barriers in the daily lives of individuals with SCIs, impacting their intention to use assistive technology in general, a prominent theme emerged focusing on the specific challenges of interacting with assistive robotics. The main theme comprises 4 subthemes: doubts about meaningfulness, uncertainty regarding reliability and safety, uneasiness about competence, and apprehension of social norms.

### Doubts About Meaningfulness

The participants reported several doubts about the meaningfulness of assistive robotics technology. Doubts about the meaningfulness of assistive robotics were expressed primarily by individuals with SCI, drawing on their own previous experiences of technologies that had promised a lot but delivered little, and were echoed by some HCPs.

A few of the participants had prior experiences of taking part in user testing of robotic devices and some had experiences with advanced technologies such as speech synthesis and Alexa. They emphasized that these technologies had not been very meaningful to use in everyday life:

*The hospital’s robotic arm I tried was large, with numerous cords and a complex setup, making it impractical for daily use. To be suitable for everyday life, I envision a more discreet, easily deployable, and user-friendly design with fewer machinery and electronic components to make it useful*.

[SCI_9]

Another participant said:

*I’ve tried using speech synthesis, but for two reasons, I’ve given up on it. Firstly, sometimes it goes well for a couple of sentences and then it turns into complete gibberish. Secondly, when constructing a text, you often don’t have all the answers in your head at the beginning. You start writing, go back, and make changes, rearrange and so on. This results in a significant amount of editing work. Reversing, erasing, and adjusting are not as straightforward with voice. Dynamically building a text doesn’t always mean having it fully clear in your head from the start.*

[SCI_1]

These quotes illustrate that the participants questioned the usefulness of advanced technologies based on their prior experience. There were also participants who doubted the usefulness of assistive robotic technologies based on what they learned from other people and through media. They highlighted that they often hear about life-changing technologies from others but doubt the truth in these stories:

*The media offer people like us [individuals with spinal cord injury] a thousand stories, and then 95% of the things are worthless*.

[SCI_3]

They did not believe what they heard due to the lack of evidence. Also, HCPs questioned the lack of evidence regarding the benefits and usefulness of assistive robotic technologies:

*I don’t think there is much scientific evidence in this regard…*

[HCP_23]

### Uncertainty Regarding Reliability and Safety

Uncertainty about reliability and safety was mentioned mainly by relatives and health care professionals, while participants with SCI themselves did not express these concerns as frequently. Relatives stressed that assistive aids need not be overly sophisticated; from their perspective, low-tech solutions appeared safer. While assistive robotics technology may offer extensive functionality, it also seemed to heighten the apprehension about its reliability and safety. One relative emphasized,

*...before it [a robot] performs a thousand functions, it has to be able to do a few basic ones in a safe and reliable way...*

[REL_9]

HCPs also underscored the importance of reliability and safety, envisioning potential mishaps that could jeopardize the well-being of individuals with SCIs. One HCP illustrated,

*Imagine it’s [a robotic arm] feeding you, it turns off and leaves you halfway with the fork in your mouth, or it’s combing your hair and leaves you with the comb tucked into the hair.*

[HCP_13]

The uncertainty surrounding reliability and safety emerged as a significant barrier to the intention to use for both relatives and HCPs.

### Uneasiness About Competence

Uneasiness about competence referred both to individuals with SCI doubting their own ability to manage complex technologies and to relatives and HCPs questioning whether potential users would be able to operate assistive robotics independently. While the participants with SCI did not raise a lot of concern about reliability and safety, as opposed to relatives and HCPs, they were instead uneasy about their own competence rather than the technologies. Almost all the participants perceived robotics as advanced and complex technology and worried that it was not for them due to their limited technological competence but also their dependence on others for most things in life. This was illustrated through their expressions, as demonstrated by

*I am no longer autonomous at all. I went from being the one who solved problems for others to not being able to do anything for anyone; now, they have to do everything for me.*

[SCI_1]

*I think that I’ve never used anything besides the wheelchair, which is manual and the crutches*.

[SCI_12]

*I don’t have any experience, I don’t use technology*.

[SCI_7]

Relatives and health care professionals were also uneasy about the competence of individuals with SCI in using assistive robotics. They drew on their own experience of helping them to handle everyday life activities as well as technology. As one of the relatives said:

*He will need another person to place or start the robotic arm... after all that would not be a solution because the person who is there would end up assisting him more than the robotic arm.*

[REL_2]

This quote illustrates how the subthemes of doubts about meaningfulness and uneasiness about competence are interrelated. If the intended user does not have the competence to use the technology by themselves, it decreases the meaningfulness of the technology.

### Apprehension of Social Norms

The subtheme, “apprehension of social norms,” highlights participants’ awareness of societal expectations and how others perceive them. Apprehension of social norms was voiced predominantly by individuals with SCI, with relatives and HCPs recognizing and sometimes sharing these concerns. Specifically, when discussing robotic limbs, many participants expressed concerns about being viewed as abnormal or strange.

They were already accustomed to being seen as “different” with 1 participant noting,

*… people may see you as if you were a robot or something like that…people already look at us with a strange face.*

[SCI_13]

The fear was that adding a robotic finger or robotic arm would further set them apart from what is considered “normal.”

HCPs and relatives shared similar reservations, expressing doubts about the societal acceptance of robotics and robotic limbs. One HCP remarked,

*I see the exoskeleton, and they look like ‘Robocop.’ Besides, who would put this device to work for them every day?*

[HCP_8]

The comment not only points to worries about social appearance but also doubts about whether the everyday effort required to set up and use assistive robotics would be perceived as worthwhile.

As the last quotation illustrates, participants reported that thoughts about robotics and robotic limbs often evoke images from movies depicting humans becoming part-robot. However, their concerns also extend to the practical aspects of using such devices, tying back to the subtheme of the perception of meaningfulness.

### Enablers

Alongside barriers, our analysis of the data revealed enablers for the intention to use assistive robotics, such as the imaginings of greater self-efficacy and independence. We interpreted the enablers as “imaginings” because they appear to be rooted in hopes and wishes for a different future. The imaginings were not specifically related to assistive robotics but rather to assistive aids in general or life in general. While the barriers described hindered participants’ intentions to use assistive robotics significantly, the participants also emphasized enablers or imagined solutions. The 2 main enabler themes: “imaginings of greater self-efficacy” and “imaginings of fulfillment of functional requirements” capture how participants envisioned assistive robotics as potentially transforming everyday life, even while simultaneously doubting whether such visions would materialize.

### Imaginings of Greater Self-Efficacy

A significant number of participants expressed a desire for increased independence and the ability to resume their pre-SCI lifestyle. While many participants had come to terms with their injury, its emotional impact remained, as they found themselves unable to lead the life they once did. Participants expressed a keen appreciation for any opportunity for improvement and assistance:

*All aids that could potentially enhance my function would be very welcome.*

[SCI_5]

Participants mostly discussed aids in general that could enhance their independence, rather than making specific reference to assistive robotics or robotic limbs. They did not specify the type of aids but rather conveyed their desires to carry out daily activities independently:

*I would like to be able to use it [a robotic limb] mainly for cleaning myself, eating, drinking, putting on shoes, dressing, that kind of thing that allows me to be more autonomous. I don’t know the possibilities, the more functions, the better. Give me as much autonomy as possible*.

[SCI_2]

The majority of the participants with SCIs expressed imaginings of having greater self-efficacy. Such imaginings could be understood as a desire to explore all possible technologies or aids for increased independence. However, the empirical data showed that they mostly used low-tech technology and perceived much technology as lacking meaningfulness to them (as illustrated above).

### Imaginings of Fulfilment of Functional Requirements

When discussing factors influencing the intention to use, “imaginings of fulfilment of requirements” emerged as one of the most significant enablers. Aids or assistive technology need to be, according to the participants, “user-friendly” and easy to learn. Participants emphasised various requirements, with statements such as

..*it is crucial to consider affordability, esthetic appeal and ease of application and maneuvering.*

[HCP_3]

*Resistance to wear and tear, absence of daily problems, ease of repair and reliable technical support are essential.*

[REL_4]

If the technology met all these requirements, then it could serve as an enabler for intention to use, according to the participants.

## Discussion

### Principal Findings

This study explored how individuals with SCI, their relatives, and HCPs in Sweden and Spain conceptualize assistive robotics in general and SRLs in particular, and which factors shape their intention to use assistive robotics.

Our findings complement previous research that has focused on specific robotic devices and their usability [52,53] and on general patterns of assistive technology adoption and abandonment [7,13,14,54-56]. Coherent with previous research, participants in our study were interested in emerging technologies that could support activities of daily living; nevertheless, they also highlighted usability, cost, and stigma as key challenges. Our contribution lies in showing how such issues play out for emerging assistive robotics such as SRLs and in demonstrating how expectations and doubts are distributed across multiple stakeholder groups.

### Relating the findings to TAM and UTAUT

Viewing our findings through the UTAUT and TAM lens [10,11], our barriers and enablers can be mapped onto core constructs while also extending them. The theme “doubts about meaningfulness” corresponds closely with performance expectancy or perceived usefulness. Thus, the participants questioned whether assistive robotics would make everyday life easier or add complexity. Subthemes of uncertainty regarding reliability, safety, and uneasiness about competence relate to effort expectancy and facilitating conditions. Hence, relatives and HCPs worried about malfunctions and safety, while individuals with SCI doubted their ability to operate assistive robotics, which were perceived as complex technologies. Apprehension of social norms directly reflects the UTUAT construct on social influence, as individuals with SCI anticipated being perceived as “too robotic” or “strange” when using visible robotic limbs in public.

The practical constraint subthemes: “navigating a changing reality,” “difficulties with awareness and access,” and “concerns about costs” speak directly to facilitating conditions. Participants highlighted limited information pathways, lack of knowledge in municipal services and financial barriers, even in subsidized health care systems like the Swedish one [50]. The findings underscore that the intention to use assistive robots is not only based on the individual attitude but shaped by broader health care system infrastructures and funding arrangements.

The enabler subtheme, “imaginaries of greater self-efficacy” and “imaginings of fulfilment of functional requirements,” align with the behavioral intention construct in TAM and UTUAT [10,11]. Participants imagined assistive robotics that were reliable, aesthetically acceptable, and well supported, and that assistive robotics would allow them to reclaim valued activities such as self-care, eating, and dressing. However, many participants also doubted that currently available or near-future assistive robotics would fully meet these requirements, suggesting a gap between positive behavioral intention and realistic expectations about actual use.

### Sociotechnical Imaginaries

Building on our reference to “sociotechnical imaginaries” in the results, we recognized the interplay between expectations and broader sociotechnical narratives, echoing discussions of the myth of technology and its advancement in the participants’ perceptions of assistive robotics. As Mayor describes in her book *“Gods and Robots: Myths, Machines and Ancient Dreams of Technology”* [57] new technologies often elicit both hope and apprehension [58,59] and can be viewed as modern myths [60,61]. The imaginaries or enablers we identified in our analysis presented a paradox. We term the tension the “meaningfulness paradox,” as participants expressed doubt about the meaningfulness of assistive robotics, their competency in handling the technology, its safety, and reliability. At the same time, they also spoke about how assistive technology could enhance self-efficacy if it met all functional requirements (which they doubted).

Thus, while TAM and UTUAT focus primarily on individual beliefs and intentions [10,11], drawing on the concepts of sociotechnical imaginaries [15-19] enables us to situate the participants' beliefs within broader collective visions of what assistive robotics could or should do. Participants' imaginings of independence, reduced caregiver burden, and improved quality of life echo existing imaginaries of robotics as a solution to workforce shortages and increasing care demands [62-64]. At the same time, the participants' narratives revealed a persistent gap between idealized expectations and lived realities, including pain, fatigue, cognitive load, and bureaucratic hurdles. For example, some participants contrasted media portrayals of robots with their own experiences of bulky robotic arms that were too demanding to set up and operate.

The tension was especially visible when participants contrasted the promise of SRLs with their experiences of earlier assistive robotic devices they had tried, which turned out to be cumbersome and unreliable. As such, sociotechnical imaginaries therefore function both as enablers for adoption, motivating interest in trying out emerging technologies, and as a potential source of disappointment if the assistive robotic device cannot live up to the expectations. Our findings suggest that managing the expectation gap is important in order to avoid future abandonment of assistive robotics, echoing previous calls for more realistic and relational approaches to social robots and care technologies [22,65].

### Implications for Research and Practice

Our findings suggest considerations for the future development of assistive robotics. They indicate that the participants’ perceptions and conceptualizations of assistive robotics were informed by ideas and sociotechnical imaginaries. The analysis underscores how idealized visions intersect with intention to use, where individuals may express a desire to use assistive robotics while simultaneously encountering barriers that complicate the translation of intent into action. Rather than framing identified barriers solely as obstacles to adoption, they can be understood as part of a broader negotiation between technical expectations and lived experiences [65]. This perspective shifts the focus from “non-compliant users” to assistive robotic systems and design that may not yet be well aligned with everyday realities of individuals with SCI and those who care for them.

For designers and engineers, our results highlight the importance of focusing on everyday meaningfulness rather than technical sophistication alone. Thus, prioritizing functions that users, relatives, and HCPs perceive as most valuable (such as self-care tasks), minimizing setup time and cognitive load. Furthermore, the subtheme “apprehension of social norms” indicates aesthetics and visibility matter. Hence, the participants did not want to look “different” or “too robotic.” As such, the design of assistive robotic devices needs to be compatible with the user’s self-perception, for example, by allowing robotic components to be concealed under clothing.

For clinicians and HCPs, our findings point to a need for clear pathways for identifying suitable candidates for assistive robotics, providing realistic information about benefits and limitations, as well as training and follow-up support. Structured introduction protocols, where assistive robotics are tried out in supervised sessions and then revisited after a trial period at home, may help individuals with SCI, relatives, and HCPs jointly assess whether the technology is useful or not. HCPs themselves also need opportunities and time to familiarize themselves with emerging technologies. Without having the time to familiarize themselves with emerging technologies, the rapid technological change of health care technologies may be perceived as additional workload instead of as support.

For policymakers and funders, ongoing concerns about costs and access underscore the need for developing reimbursement models and procurement strategies that make assistive robotics financially accessible, while also ensuring that funding covers training and maintenance. Models that support periodic reassessment may be needed to match the fluctuating needs of people with SCI. Involving people with SCI, their relatives, and HCPs in priority-setting and evaluation processes may help align investments with what is perceived as useful in everyday life.

As evidenced by numerous studies, the process of adopting new technology is far from linear [16,66,67]. Individuals’ perceptions and intentions to either embrace or reject a technology are not static; rather, they are dynamic, continually shaped and negotiated as people interact with the technology [68]. Our results suggest that the ways individuals with SCI engage with assistive technologies are contingent on their lived experiences, evolving needs, and changing technological landscape. Overhyping the capabilities of assistive robotics risks fostering unrealistic expectations that could shape disengagement and disappointment [69]. Rather than focusing on promotional narratives, there is value in exploring how assistive robotics are experienced and imagined within participants’ everyday lives. As researchers, we can contribute by facilitating discussions that reflect both possibilities and limitations, offering a grounded perspective on what assistive robotics can achieve and the type of support they can provide [65].

### Limitations of the Study

A limitation of this study is that the inclusion and exclusion criteria did not take into account participants’ time since injury, prior use of assistive technology, or whether they had completed comprehensive rehabilitation. The sampling strategy was a deliberate choice intended to invite a diverse range of perspectives and lived experiences, rather than predefining the sample based on rehabilitation history or technological exposure. However, we acknowledge that prior rehabilitation experiences and the use of assistive technologies may have shaped participants’ attitudes toward adopting new assistive robotics solutions. Thus, the findings reported in the paper pertain to individuals with SCIs, constituting a highly heterogeneous group. Rather than viewing heterogeneity as a limitation alone, heterogeneity among the participants hopefully brought a more layered understanding of varying expectations, needs, and concerns surrounding assistive robotics. Nonetheless, the study has been strengthened by involving individuals from 2 different countries, as well as including relatives and HCPs. Therefore, the findings are likely to be beneficial for developers, practitioners, and researchers working on projects with similar technologies and characteristics for the target group.

Another limitation, but also an interpretive choice, was the use of the UTAUT as a lens when discussing the findings. The conceptual framings were used to help interpret and frame overarching patterns from the findings in the discussion. The data were first analyzed inductively, seeking all themes without being led by the need to fit participants’ perceptions into a certain framework. As such, the interplay between emergent interpretation and structured theory did not impose a rigid framework but instead facilitated a dynamic engagement when discussing the findings, allowing both participant-driven insights and established conceptual framings to emerge. The interpretive approach was conducted with attention to maintaining the rigor of qualitative research. The recurring patterns observed in the data suggest not only coherence but also shared meanings among participants. Even so, using TAM and UTAUT may foreground certain aspects of technology acceptance while overlooking others. Future research could draw on alternative frameworks.

An additional limitation of the study was that the participants expressed gratitude for participating in the interviews and workshops, which may result in overly positive attitudes toward assistive robotics. However, as seen in the results, the data revealed multiple barriers to the intention to use, as interpreted in our analysis, which suggests that expressions of gratitude did not necessarily equate to uncritical acceptance of assistive robotics but coexisted with articulated concerns and constraints.

### Conclusions

This study shows that intentions to use assistive robotics among individuals with SCI, their relatives, and HCPs are shaped by a combination of barriers and enablers. The findings revealed that the barriers were clustered around practical constraints (such as navigating new realities, fluctuating health, limited awareness and access, and concerns about costs) and interaction with assistive robotics (including doubts about meaningfulness, uncertainty regarding reliability and safety, uneasiness about competence, and apprehension of social norms). Individuals with SCI described fluctuating pain, fatigue, and transition periods after discharge as making it difficult to invest energy in learning and using assistive robotics, while HCPs highlighted how continuous technological change increased their workload and required repeated training. Difficulties with awareness and access were mainly voiced by individuals with SCI and their relatives, who experienced fragmented information and limited support, whereas healthcare professionals perceived a wide range of available aids that were nevertheless seldom prescribed. Concerns about costs were shared by all 3 stakeholder groups and were seen as a major barrier to the intention to use assistive robotics.

The theme of *“*interaction with assistive robotics” revealed doubts about meaningfulness, uncertainty about reliability and safety, uneasiness about competence, and apprehension of social norms. Individuals with SCI tended to question their own ability to manage complex technologies such as assistive robotics in everyday life, while relatives and HCPs questioned whether assistive robotics would reduce dependence. Apprehension of social norms was voiced predominantly by individuals with SCI, who feared that visible robotic limbs would reinforce perceptions of them as “different.” At the same time, participants’ imaginings of greater self-efficacy and fulfillment of functional requirements, often expressed as hopes of regaining pre-injury abilities and reducing dependence on others, functioned as important enablers across groups. We conceptualized the pattern of ambivalence as a meaningfulness paradox, as participants expressed doubts about the meaningfulness of assistive robotics in everyday life, their own competence in handling assistive robotics, and their safety and reliability. Nonetheless, they also spoke about how assistive robotics could enhance self-efficacy if assistive robotics met all functional requirements, a circumstance they often doubted. The results suggest that assistive robotics should be understood in relation to the complexities of everyday life with SCI, caring relationships, and the imaginaries that shape how emerging technologies are perceived.

## Supplementary material

10.2196/72080 Multimedia Appendix 1 Guidelines for in-depth workshop with individuals with a spinal cord injury.

10.2196/72080 Multimedia Appendix 2 Interview guide for patients.

10.2196/72080 Multimedia Appendix 3 Interview guide for relatives.

10.2196/72080 Multimedia Appendix 4 Interview guide for health care professionals.

10.2196/72080 Multimedia Appendix 5 Guidelines workshops with health care professionals.

## Abbreviations

SCI: spinal cord injury
SRL: supernumerary robotic limb
TAM: Technology Acceptance Model
UTAUT: Unified Theory of Acceptance and Use of Technology
