# Supplementary material for: Exploring Barriers and Enablers for the Intention to Use Assistive Robotics Among People With Spinal Cord Injury and Those Involved in Their Care: Qualitative Study
Source: JMIR Rehabil Assist Technol. 2026 Feb 17;13:e72080. doi: 10.2196/72080 (PMC12912652; doi:10.2196/72080)
Supplement: Multimedia Appendix 2 [file rehab-v13-e72080-s002.pdf]

Interview guide for patients

Interviewees: Patients who have had a spinal cord injury.

Please remember: Use probes for each question. For example: can you elaborate on what you mean by xx? Can you give examples of xx? What do you feel when xx happens? How often does xx happen? You say xx, what do you mean?

It is important to use probes for each question in order to get in-depth answers.

Questions, not mentioned below, that arise during the interview should also be discussed (if they concern " assistive robotic technology " or patients' needs and wishes) – use probes to dig deep into the issue.

Important to do not introduce the technologies in the HARIA project until it says so in the interview guide.

Interview

1. When did you have a spinal cord injury?
2. Would you like to describe what impact the spinal cord injury has had on your everyday life? In relation to others? In relation to ADL?
3. How has the spinal cord injury affected your life?
4. What kind of support do you benefit from?
5. What kinds of movements or activities do you have trouble performing? What obstacles do you have in your everyday life?
6. Are there aids that help you with the movements/activities mentioned?
7. What support is available and works well? Which aids/ assistive technology are available and work well?
8. What support is missing? What aids/assistive technology are missing?
9. What is your experience with the use of digital aids/assistive technologies?
10. What kinds of aids/assistive technologies do you have experience using? Do you have experience with "assistive robotic technology"?
11. Can you describe what the process looks like when you get a new aid/assistive technology? Who is involved? Which actors are important to know?
12. Who is responsible for showing and teaching how to use the aid/assistive technology?
13. Who is responsible for the purchase of aids/assistive technology?

    (The interviewer describes what "assistive robotic technology" is (based on the technologies in the HARIA project) and what they can be used for - unless the interviewee has experience/knowledge of "assistive robotic technology")

14. If you get to participate and influence, what would you like "assistive robotic technology" to be used for in your everyday life? Any ideas on function/functionality? Design? What are your needs? Wishes?
15. What kind of benefits can you see with assistive robotic technology? Disadvantages?
16. How do you think "assistive robotic technology" can change your everyday life? Benefits? Disadvantages?

Closure

28 Is there anything else you can think of regarding assistive robotic technology that you think we haven't covered?
