# Supplementary material for: Exploring Barriers and Enablers for the Intention to Use Assistive Robotics Among People With Spinal Cord Injury and Those Involved in Their Care: Qualitative Study
Source: JMIR Rehabil Assist Technol. 2026 Feb 17;13:e72080. doi: 10.2196/72080 (PMC12912652; doi:10.2196/72080)
Supplement: Multimedia Appendix 3 [file rehab-v13-e72080-s003.pdf]

Interview guide for Relatives

Interviewees: Relatives/relatives of people who have a spinal cord injury. X below refers to the individual (next of kin) with a spinal cord injury

Please remember: Use probes for each question. For example: can you elaborate on what you mean by xx? Can you give examples of xx? What do you feel when xx happens? How often does xx happen? You say xx, what do you mean?

It is important to use probes for each question in order to get in-depth answers.

Questions, not mentioned below, that arise during the interview should also be discussed (if they concern " assistive robotic technology " or patients' needs and wishes) – use probes to dig deep into the issue.

Important to do not introduce the technologies in the HARIA project until it says so in the interview guide.

Interview

1. What is your role/relationship to X with spinal cord injury?
2. How long has X had a spinal cord injury?
3. Would you like to describe what impact the spinal cord injury has had on your relationship?
4. How has the spinal cord injury affected X's life?
5. What kind of support do you benefit from? You? X?
6. What support is available and works? For you? For X?
7. What support is missing? For you? For X?
8. What kinds of movements or activities does X have trouble performing? What obstacles does X have in his everyday life?
9. Are there aids that help X in the mentioned movements/activities?
10. What is your experience with X use of digital aids/assistive technology?
11. What kinds of aids/assistive technology do you have experience using? Do you have experience with smart "assistive robotic technology" ?
12. Can you describe what the process looks like when X gets a new aid? Who is involved? Which actors are important to know?
13. Who is responsible for showing and teaching how to use the aid?
14. Who is responsible for the purchase of aids?

    (The interviewer describes what smart " assistive robotic technology" is (based on the technologies in the HARIA project) and what they can be used for – unless the interviewee has experience/knowledge of smart " assistive robotic technology ''

15. If you get to be involved and influence, what would you like to smart assistive robotic technology could be used for when it comes to X and others who have a spinal cord injury/have had a stroke? What do you think X needs? Wishes?
16. What kind of benefits can you see with assistive devices (smart assistive robotic technology)? Disadvantages?
17. How do you think smart assistive robotic technology can change X everyday life?
18. In what way do you think that smart assistive robotic technology will change your relationship? Benefits? Disadvantages?

Closure

28 Is there anything else that you will think of that relates to smart assistive robotic technology that you think we haven't covered?
