# Supplementary material for: Exploring Barriers and Enablers for the Intention to Use Assistive Robotics Among People With Spinal Cord Injury and Those Involved in Their Care: Qualitative Study
Source: JMIR Rehabil Assist Technol. 2026 Feb 17;13:e72080. doi: 10.2196/72080 (PMC12912652; doi:10.2196/72080)
Supplement: Multimedia Appendix 4 [file rehab-v13-e72080-s004.pdf]

Interview guide for healthcare professionals

Interviewees: Professionals (nurses, doctors, occupational therapists, etc.) in healthcare who work with people who have a spinal cord injury.

Please remember: Use probes for each question. For example: can you elaborate on what you mean by xx? Can you give examples of xx? What do you feel when xx happens? How often does xx happen? You say xx, what do you mean?

It is important to use probes for each question in order to get in-depth answers.

Questions, not mentioned below, that arise during the interview should also be discussed (if they concern " assistive robotic technology " or patients' needs and wishes) – use probes to dig deep into the issue.

Important to do not introduce the technologies in the HARIA project until it says so in the interview guide.

Interview

1. What is your role/occupation?
2. How long have you worked as ' nurse/doctor/occupational therapist/other'?
3. Would you like to describe your work to me?
4. How many patients do you handle who have a spinal cord injury during a work week?
5. What kind of support do patients need?
6. What support is available and works?
7. What support is missing?
8. What kinds of movements or activities do your patients have trouble performing? What obstacles do they (the patients) have in their everyday life?
9. Are there aids that help them in said/above mentioned movements/activities?
10. What is your experience of using digital aids in your care of patients who have a spinal cord injury stroke?
11. What kinds of aids do you have experience using? Do you have experience with " assistive robotic technology ?
12. Can you describe what the process looks like when a patient is to receive a new aid? Who is involved? Which actors are important to know?
13. Who is responsible for showing and teaching how to use the aid?
14. Who is responsible for the purchase of aids?

(The interviewer describes what " assistive robotic technology " is (based on the technologies in the HARIA project) and what they can be used for - unless the interviewee has experience/knowledge of " assistive robotic technology ''

15. If you get to be involved and influence, what would you like ” assistive robotic technology ” could be used for when it comes to your patients? What do you think they need? Wishes?
16. What kind of benefits can you see with assistive devices (" assistive robotic technology ”)?
17. What kind of disadvantages can you see with assistive devices (" assistive robotic technology ”)?
18. How do you think that ” assistive robotic technology ” can change patients' everyday life?
19. In what way do you think that ” assistive robotic technology ” will change your work? Benefits? Disadvantages?

Closure

28 Is there anything additional that you will think of regarding " assistive robotic technology ” that you think we haven't covered?
