# Supplementary material for: Exploring Barriers and Enablers for the Intention to Use Assistive Robotics Among People With Spinal Cord Injury and Those Involved in Their Care: Qualitative Study
Source: JMIR Rehabil Assist Technol. 2026 Feb 17;13:e72080. doi: 10.2196/72080 (PMC12912652; doi:10.2196/72080)
Supplement: Multimedia Appendix 5 [file rehab-v13-e72080-s005.pdf]

Guidelines workshops with healthcare professionals

AIM

The aim of the workshops is to gather insights on the participants experiences and opinions on everyday life with spinal cord injury, and to getter understandings of their perception, attitudes and motivations.

1. The first part of the workshop focusses on social issues and current aids/assistive technology.

2. The second part is developed to better understand user requirements and to identify opportunities.

Step-by-step guide for conducting workshops

1) Start the power point presentation

2) Slide 1: Introduce yourself (one person act as moderator and another person to take notes), explain the purpose of the workshop, and how the interviewees insights will contribute to the project. Explain that the goal of the workshop is to identify the hindrances and opportunities their patients face in their daily lives as a result of their spinal cord injury as well as on available aids/assistive technologies and user needs and requirements for future aids/assistive technology.

3) Obtain inform consent

4) Slide 2: Introduce the agenda. Ensure that you create a comfortable and conducive environment for the participants. Start with a warm-up activity where each person gets their chance to introduce themselves. Ask about their professional background and experiences (years in the profession), and how they became interested in partaking in the workshop

5) Slide 3: Give participants a few minutes to brainstorm the opportunities and possibilities that their patients have in their daily lives despite their spinal cord injury. Have them write down these opportunities on sticky notes or index cards.

6) Slide 4:

    a. Once everyone has had a chance to brainstorm, have them share their opportunities one at a time and place them on a whiteboard or a flip chart.

b. After everyone has shared their opportunities, facilitate a group discussion to identify common themes and positive experiences.
c. Encourage participants to share their successes and positive experiences, and ask questions to clarify and explore the opportunities
d. Take photos of the whiteboard or flip chart for documentation

7) Slide 5: Give participants a few minutes to individually brainstorm all the things that they know their patients find <u>difficult or limiting</u> in their daily lives due to their spinal cord injury. Ask them to write down these hindrances on sticky notes or index cards.

8) Slide 6:
    a. Once everyone has had a chance to brainstorm, have them share their identified hindrances one at a time and place them on a whiteboard or a flip chart.
    b. Group discussion: After everyone has shared their identified hindrances, facilitate a group discussion to identify common themes and issues. Encourage participants to share their experiences and challenges, and ask questions to clarify and explore the hindrances.
    c. take photos of the whiteboard or flip chart for documentation

9) Slide 7: Give participants a few minutes to brainstorm the aids and assistive technologies their patients have at home and the motivations for use and non-use - **specially in regards to their arms and hands**
Ask them to write it down om sticky notes

10) Slide 8:
    a. Once everyone has had a chance to brainstorm, have them share their insights on available aids/assistive technologies that their patients use, one at a time, and place them on a whiteboard or a flip chart. After everyone has shared their insights, facilitate a group discussion and encourage participants to share their experiences, and ask questions to clarify and explore usage, as well as similarities and differences
    b. take photos of the whiteboard or flip chart for documentation

c. Next let everyone, one by one, share they the sticky notes on aids their patients have at home but do not use, and place them on a whiteboard or a flip chart. Ask questions about the reasons for non-use. After everyone has shared their insights, facilitate a group discussion and encourage participants to share their experiences, and ask questions to clarify and explore non-usage, as well as similarities and differences

d. take photos of the whiteboard or flip chart for documentation

11) BREAK – coffee and refreshment

Part 2: Now it is time to start to elicit user requirements and opportunities of technical aids. This is done by looking at and discuss a few different versions of technical aids (one at a time). Interesting aspects to discuss is for example (but is not limited to) functionality, easiness in taking on/off, aesthetics, flexibility, time and effort (how long do you think patients will wear it and how much time/effort does it require to put on). Let the participants guide the discussion. The things that come up in the discussions are helpful to understand and define user requirements.

12) Slide 10: The first product is made to assist a person to grip, hold and release objects by pulling artificial tendon - like a puppeteer. Show video: https://www.youtube.com/watch?v=KskES39OE6M

    Facilitate a group discussion to identify common themes and issues about the product. Ask for example: "What are you thinking?" "What do you think would make your patients use this product? "In which situations?"

    Encourage participants to share their impression and insights, and ask questions to clarify and explore their ideas

    Take extensive notes on what is being said and of who

13) Slide 11: The second example is a sixth finger. The sixth finger is a wearable robotic device to assist or augment the human grasping capabilities. The device can be tied to different parts of the human body, such as the forearm, wrist or ankle, and when unused fits onto the body, wrapping the limb or body part on which it is worn (like a bracelet, anklet, belt). Show video: https://www.youtube.com/watch?v=MqGFQX1axBE

Facilitate a group discussion to identify common themes and issues about the product. Ask for example: "What are you thinking?" "What do you think would make your patients use this product? "In which situations?"

Encourage participants to share their impression and insights, and ask questions to clarify and explore their ideas

Take extensive notes on what is being said and of who

14) Slide 12: The third example is a single robotic arm placed on a fixed or mobile base (possibly wheelchair mounted). The arm performs both interaction with the environment (e.g., manipulate, and use objects in the peripersonal space of the user, without contact with the user) and actions that involve physical interaction with the user (e.g., grooming, feeding).

    Facilitate a group discussion to identify common themes and issues about the product. Ask for example: "What are you thinking?" "What do you think would make your patients use this product? "In which situations?"

    Encourage participants to share their impression and insights, and ask questions to clarify and explore their ideas

    Take extensive notes on what is being said and of whom

15) Slide 13: The fourth example is a dual-arm robotic system with fixed or mobile base is used to carry out different operations, from grasping and moving large objects to performing bimanual tasks. Facilitate a group discussion to identify common themes and issues about the product. Ask for example: "What are you thinking?" "What do you think would make your patients use this product? "In which situations?"

    Encourage participants to share their impression and insights, and ask questions to clarify and explore their ideas

    Take extensive notes on what is being said and of whom

16) End the workshop by summarizing the key takeaways and thanking the participants for their participation. Also, ask the participants if they would like to be contacted for future workshops, interviews, etc
